# Collagen Extracted from Bigeye Tuna (*Thunnus obesus*) Skin by Isoelectric Precipitation: Physicochemical Properties, Proliferation, and Migration Activities

**DOI:** 10.3390/md17050261

**Published:** 2019-05-01

**Authors:** Xinhui Lin, Yinyue Chen, Huoxi Jin, Qiaoling Zhao, Chenjuan Liu, Renwei Li, Fangmiao Yu, Yan Chen, Fangfang Huang, Zuisu Yang, Guofang Ding, Yunping Tang

**Affiliations:** 1 Zhejiang Provincial Engineering Technology Research Center of Marine Biomedical Products, School of Food and Pharmacy, Zhejiang Ocean University, Zhoushan 316022, China; linxinhui1995@163.com (X.L.); zhdchenyingyue@163.com (Y.C.); jinhuoxi@163.com (H.J.); 17805800624@163.com (C.L.); fmyu@zjou.edu.cn (F.Y.); gracegang@126.com (F.H.); abc1967@126.com (Z.Y.); dinggf2007@163.com (G.D.); 2 Zhoushan Institute for Food and Drug Control, Zhoushan 316000, China; zql850410@126.com; 3 Zhejiang Ocean Family CO., LTD, Zhoushan 316022, China; lirw6886@163.com; 4 Zhejiang Changxing Pharmaceutical CO., LTD, Huzhou 313108, China

**Keywords:** *Thunnus obesus*, collagen, isoelectric precipitation, physicochemical properties, proliferation and migration

## Abstract

Collagen was extracted from bigeye tuna (*Thunnus obesus*) skins by salting-out (PSC-SO) and isoelectric precipitation (PSC-IP) methods. The yield of the PSC-IP product was approximately 17.17% (dry weight), which was greater than the yield obtained from PSC-SO (14.14% dry weight). Sodium dodecyl sulfate-polyacrylamide gel electrophoresis analysis indicated that collagen from bigeye tuna skin belongs to collagen type I. Inductively coupled plasma mass spectrometry results indicate that the heavy metal abundance in PSC-IP was lower than the maximum acceptable amounts according to Chinese regulatory standards. In addition, results from a methylthiazolyldiphenyl-tetrazolium bromide assay and an in vitro scratch assay demonstrated that PSC-IP could promote the proliferation and migration of NIH-3T3 fibroblasts. Overall, results suggest PSC-IP could be used to rapidly extract collagen from marine by-products instead of traditional salting-out methods. Collagen from bigeye tuna skin may also have strong potential for cosmetic and biomedical applications.

## 1. Introduction

Rapid developments of seafood products in the marine product processing industry have led to a large number of marine by-products being discarded without treatment, such as fish skin, bones, scales, and swimming bladders [1,2]. By-products are a cause of environmental issue and are wastes of biological resources, therefore the utilization of by-products to produce high-added-value compounds is an urgent problem, such as the blue granary scientific and technological innovation plan in China. Collagen, which accounts for approximately thirty percent of the total protein content, is the most abundant fibrous protein in animals [3,4]. Recently, marine collagen has been extracted from sponges, octopus, jellyfishes, squids, and fish offal such as skins, bones, fins, and scales [5,6,7,8]. Marine collagen has attracted an increasing interest for applications in the biomedical, and pharmaceutical, cosmetic and food industries, as it has no religious limitations, and shows low immunogenicity and non-cytotoxicity [9,10,11]. Therefore, the extraction of collagen from marine by-products would be a suitable way to utilize them, which could produce a high-added-value compound. 

Neutral salt solubilization, acid solubilization, and pepsin solubilization are three major methods for extracting collagen in the collagen extraction phase [6]. However, the recovery phase is also an extremely important step during industrial collagen production. The salting-out method was often used for precipitating collagen during the recovery process [12,13,14]. The main principle of the salting-out method is that the charge carried by salt ions in solution neutralizes the surface charge of collagen molecules, which decreases the electrostatic interactions between collagen molecules and leads to their gradual precipitation. The salting-out method offers high recovery rates of collagen and does not affect its triple helix structure; therefore, it has often been used in previous studies [14,15]. However, the addition of high concentrations of salt can greatly prolong the downstream dialysis process. Generally, it takes three to four days of dialysis after salting-out to ensure that the final product is not affected by the salt and acid [3,15]. Moreover, dialysis of collagen extract containing a high concentration of salt produces a large amount of wastewater. Therefore, the purification process could be improved by the development of a highly efficient, highly productive, and sustainable method to take full advantage of collagen-rich fish by-products.

Isoelectric precipitation has become a popular method for protein purification due to its high efficiency and strong specificity; it also does not often require a lengthy downstream dialysis for the product [16,17,18]. Isoelectric precipitation is based on the principle that proteins have their lowest solubility at their isoelectric point and that different proteins have different isoelectric points. Isoelectric precipitation has been used for the separation and purification of proteins such as PSE-like chicken protein [19], walnut protein [20], Chinese quince seed protein [21], and ovalbumin [22]. However, there are few reports demonstrating the use of isoelectric precipitation for the recovery and purification of collagen. 

Tuna are an economically important fish worldwide; in 2018 the estimated global harvest of tuna was 7.5 million tons [23]. Because the white meat of tuna is only used for sashimi or canned, the tuna industry produces a lot of waste or by-products, which includes fish heads, bones, skins, scales, internal organs, and dark meat, which account for approximately 50% to 70% of the total mass [24]. However, there are no reports of the use of isoelectric precipitation to extract collagen from bigeye tuna (*Thunnus obesus*) skins. Therefore, the aim of this study is to use isoelectric precipitation to extract collagen from bigeye tuna skin. The physiochemical properties of collagen obtained from isoelectric precipitation, and its activity on NIH-3T3 proliferation and migration are also evaluated to assess its suitability for biomedical and cosmetic applications

## 2. Results and Discussion

### 2.1. Determination of the Isoelectric Point of Collagen

**Figure 1:**
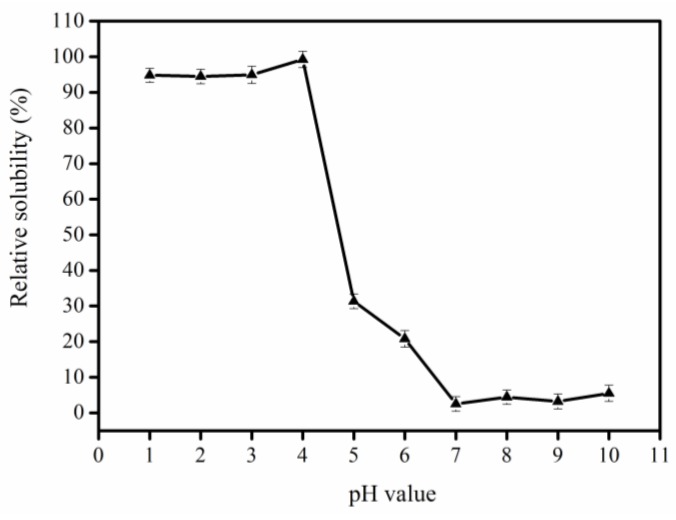
Determination of the isoelectric point of pepsin-solubilized collagen (PSC) from bigeye tuna skin.

The determination of the collagen isoelectric point is necessary for using isoelectric precipitation to recover and purify collagen. As shown in Figure 1, pepsin-solubilized collagen (PSC) from bigeye tuna skin had the best solubility in the range of acidic pH values (≤4.0) and had lower solubility in the range of neutral or alkaline pH values. Similarity, the PSC from *Nibea japonica* skin and the PSC from silver carp showed the maximum solubility in the pH range of 1.0–4.0, and lower solubility in the neutral or slightly alkaline pH range [9,25]. Our result was consistent with these studies. When the pH value is above or below the isoelectric point of a protein, the net charge, repulsive forces, and interaction capacity with water, increase. However, if the protein has no net charge at the isoelectric point, the protein will aggregate and precipitate due to hydrophobic–hydrophobic interactions. In the present study, PSC showed the lowest solubility at pH 7.0, therefore, this value was selected for collagen extraction from bigeye tuna skin. 

### 2.2. Collagen Yield from Bigeye Tuna Skin

**Figure 2:**
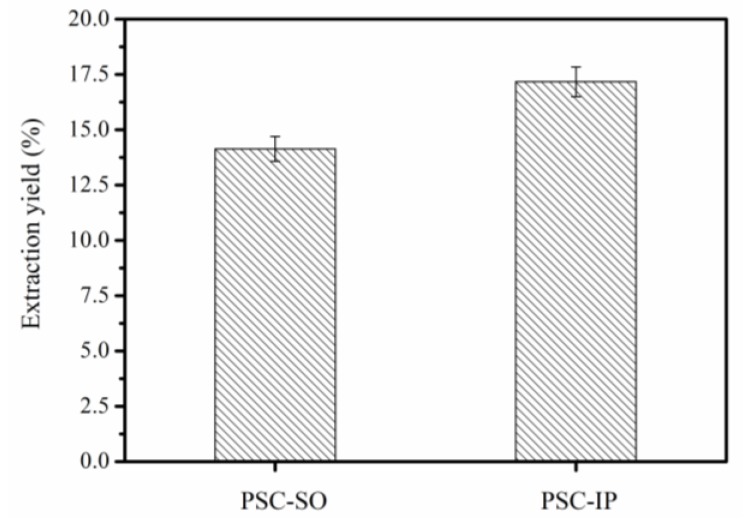
Comparison of extraction yield of collagen by the salting out method and isoelectric precipitation method (dry weight).

The PSC from the bigeye tuna skin was precipitated using the salting-out method (PSC-SO) and isoelectric precipitation method (PSC-IP) separately. The yield of PSC-SO and PSC-IP was 14.14% (dry weight) and 17.17% (dry weight), respectively (Figure 2). Due to the high concentration of NaCl (1.5 M) in PSC-CO, it took four days of dialysis after salting-out to ensure that PSC-SO was not affected by NaCl and acetic acid. In addition, large amounts of wastewater were produced due to the use of high salt concentrations. Our results were consistent with previous studies that demonstrated the use of NaCl (0.5–1.5 M) to precipitate collagen [3,15]. Due to the low salt concentration required for PSC-IP, dialysis only took two days after the process and less waste water was produced than for PSC-SO. Since collagen yields were greater from PSC-IP, and a shorter time of dialysis was required than for PSC-SO, the PSC-IP method was selected for extracting PSC from bigeye tuna skin. 

### 2.3. Sodium Dodecyl Sulfate-Polyacrylamide Gel Electrophoresis (SDS-PAGE) Analysis

**Figure 3:**
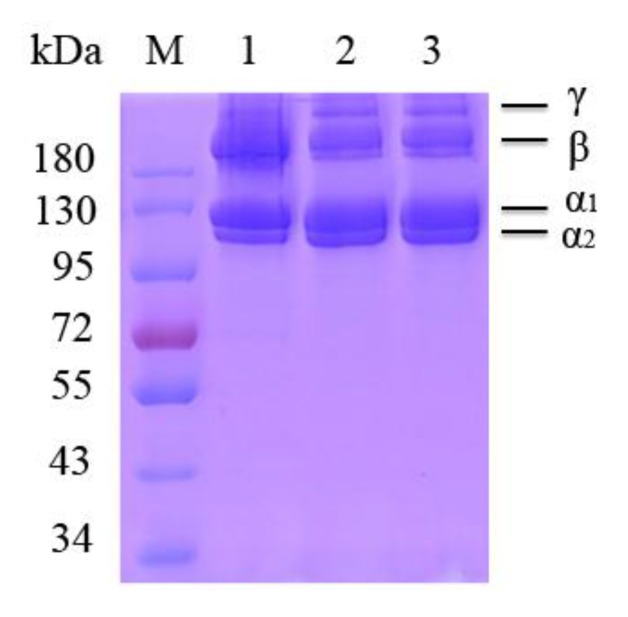
Sodium Dodecyl Sulfate-Polyacrylamide Gel Electrophoresis (SDS-PAGE) analysis of PSC-SO and PSC-PI product from bigeye tuna skin. M: Protein markers; Lane 1: Bovine collagen type I; lane 2: PSC-SO from bigeye tuna skin; lane 3: PSC-PI from bigeye tuna skin.

Figure 3 indicates the SDS-PAGE patterns of PSC-SO and PSC-PI products extracted from bigeye tuna skin, along with bovine collagen type I for comparison. The band patterns of PSC-SO and PSC-PI were highly identical, which contained two clear bands attributed to two different types of α-chains (α_1_ and α_2_) in accordance with bovine collagen type I (Figure 3). The density of α_1_-chain band was approximately 2-fold greater than α_2_-chain band, indicating that collagen type I was the main collagen in bigeye tuna skin. In addition, high molecular weight compounds, including β compounds as well as a small number of γ compounds were also observed in PSC-SO and PSC-PI products. Ahmed et al. [26] explored bacterial collagen protease to extracted collagen from bigeye tuna, and their SDS-PAGE results indicated that collagen from bigeye tuna consisted of two different types of α-chains (α_1_ and α_2_), which was consistent with our result. Our results were also consistent with previous results from PSC extracted from other marine fish skins, including *Scomberomorous niphonius* [27], *Evenchelys macrura* [28], and *Aluterus monocerous* [29], which also belongs to collagen type I. 

### 2.4. Amino Acid Contents

**Figure 4:**
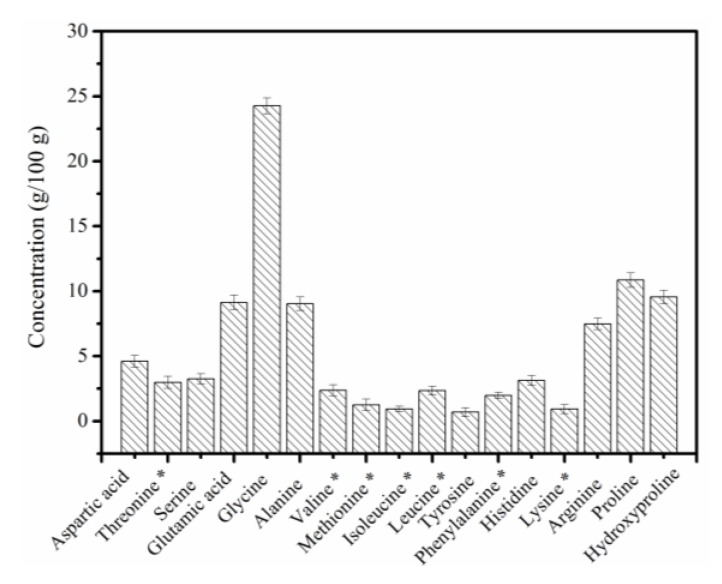
Amino acid contents of PSC-IP product extracted from skin of bigeye tuna. Note: * Essential amino acid.

The amino acid contents of the PSC-IP product are shown in Figure 4. The major amino acid in the PSC-IP product is glycine, which constitutes approximately 24% of the total amino acid contents. Other amino acids that were in high proportions were proline (10.86%), hydroxyproline (9.56%), alanine (9.04%), glutamic acid (9.13%), and arginine (7.49%). Cysteine was not detected in the PSC-IP product from bigeye tuna skin. The amino acid contents in PSC-IP product were consistent with the collagen from bigeye tuna in the previous study (glycine (22.2–22.7%), proline (14.8–15.1%), alanine (9.7–9.9%), glutamic acid (9.8–9.9%), hydroxyproline (8.0–8.2%)) [26]. In addition, the high content of glycine, proline, and hydroxyproline in the PSC-IP product is consistent with the high frequency of occurrence of the glycine-proline-hydroxyproline sequence in collagen, which is essential for triple helical formation [4,5]. The content of amino acids (proline and hydroxyproline) in PSC-IP product is 20.42%, which was similar to jellyfish *Acromitus hardenbergi* collagen (19.50%) [2], crap scales or bones collagen (19.2%) [30]. 

### 2.5. Fourier Transform Infrared Spectroscopy (FTIR) Analysis

**Figure 5:**
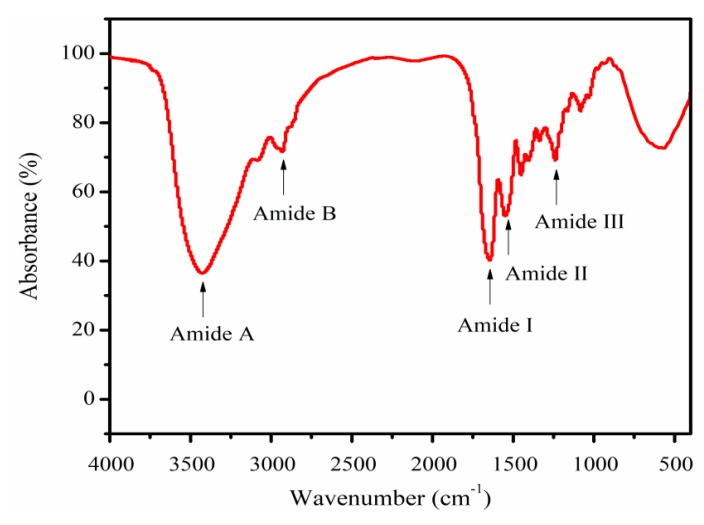
FTIR spectra of PSC-IP product from bigeye tuna skin.

Figure 5 indicates the FTIR spectra of the PSC-IP product from bigeye tuna skin. The five main absorption bands of the PSC-IP product were located in the amide zone, containing a peak for amide A (3425. 57 cm^−1^), B (2930.97 cm^−1^), I (1646.26 cm^−1^), II (1550.75 cm^−1^), and III (1238.94 cm^−1^). The amide A wavenumber of the PSC-IP product was located at 3425.57cm^−1^, which fits the common wavenumber of free N–H vibrations as an indication of hydrogen bonds [31]. The wavenumber of the amide B band of the PSC-IP product was 2930.97 cm^−1^, indicating the existence of the asymmetrical stretch of CH_2_. The amide I band is associated with C=O stretching vibration on the main polypeptide chain or the hydrogen bond coupled with COO^-^ [4]. The amide I band from the PSC-IP sample is supported by strong absorbance shown in the range of 1600–1700 cm^−1^. The amide II band represents the N–H bending vibration couples with C–N stretching vibration, and the measured wavenumbers of PSC-IP are within the range of 1550–1600 cm^−1^. In addition, the appearance of the PSC-IP sample amide III band corresponds to the helical arrangement in the PSC sample.

### 2.6. Inductively Coupled Plasma Mass Spectrometry (ICP-MS)

Heavy metal ions such as As, Pb, and Hg were often used for evaluating the extracted collagen for cosmetic and biomedical applications [4,14]. For example, Tang et al. [4] used ICP-MS to detect As, Pb, and Hg content in collagen from *Nibea japonica* skin and found the content of these heavy metal ions was significantly lower than Chinese regulatory standards. Zhang et al. [14] used ICP-MS to detect these heavy metal ions in collagen from frog skin, which was also lower than Chinese regulatory standards. Thus, the contents of As, Pb, and Hg in PSC-IP product were analyzed using ICP-MS to verify the possibility of applying PSC-IP for cosmetic and biomedical applications (Table 1). ICP-MS results indicate that the heavy metal abundance in PSC-IP was lower than the maximum acceptable amounts according to Chinese regulatory standards (GB 6783-2013). Our results indicate that the heavy metal ions As, Pb, and Hg did not accumulate during the PSC-IP extraction process. Thus, the PSC-IP process is safe for use on bigeye tuna skin for cosmetic and biomedical applications. 

### 2.7. Cytotoxic and Allergenic Tests

**Figure 6:**
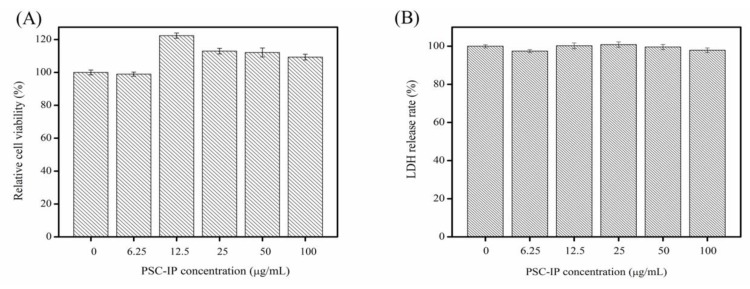
Relative cell viability (**A**) and lactate dehydrogenase (LDH) release (**B**) as affected by 24 h treatment of PSC-IP product (0, 6.25, 12.5, 25, 50, and 100 μg/mL) from bigeye tuna skin.

The cytotoxicity and sensitization of the PSC-IP product from bigeye tuna skin were detected using a methylthiazolyldiphenyl-tetrazolium bromide (MTT) assay and lactate dehydrogenase (LDH) toxicity assay. The MTT assay was used to assess the cell compatibility of the PSC-IP product, and results are shown in Figure 6A. Results indicate that after treatment with increasing concentrations of the PSC-IP product, the viability of NIH-3T3 fibroblasts did not decrease after 24 h incubation. In addition, the PSC-IP product promoted the growth of NIH-3T3 fibroblasts. Therefore, results demonstrate that collagen from bigeye tuna skin has no significant cytotoxic effect in vitro. Our finding agreed with that of Jeong et al. [32], where pre-osteoblast (MC3T3-E1) cells were used for biocompatibility evaluation of collagen from *Thunnus obesus* bone. Their results revealed that collagen scaffolds from *Thunnus obesus* bone were biocompatible and non-toxic in vitro. The cytoplasm in every human tissue contains LDH, and the disruption of cell membrane integrity leads to an increase in LDH concentration in the surrounding matrix. Thus, due to its close association with allergic reactions and inflammation, LDH release has been utilized as a criterion to evaluate allergenicity [33,34]. As shown in Figure 6B, the LDH release of cells in the presence of the PSC-IP product was relatively low when compared with untreated cells. The results indicate that PSC-IP extract from bigeye tuna skin could be considered as a non-cytotoxic and hypoallergenic biomaterial for cosmetic and biomedical applications.

### 2.8. Morphological Examination

**Figure 7:**
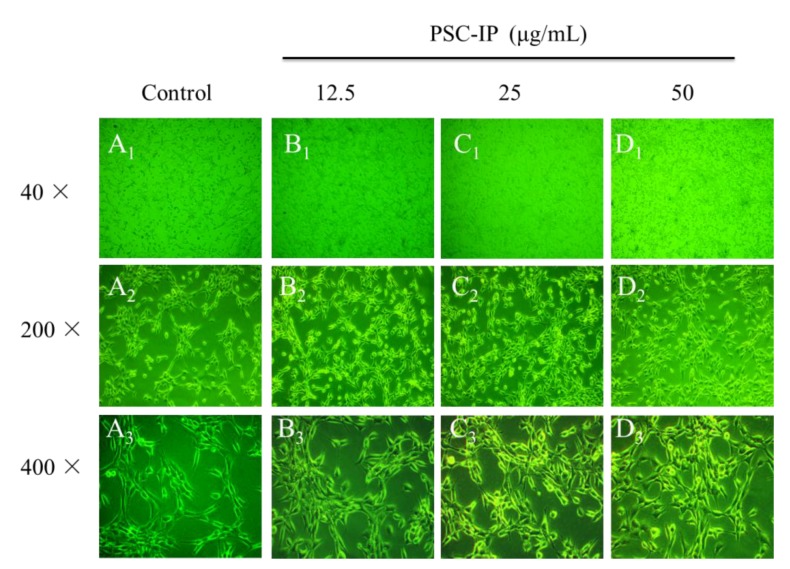
Morphological changes of NIH-3T3 cells treated with 0, 12.5, 25, and 50 μg/mL of PSC-IP product from bigeye tuna skin, respectively (40×, 200×, 400×). **A_1_**–**A_3_:** Untreated cells; **B_1_**–**B_3_:** Treated cells with 12.5 µg/mL of PSC-IP; **C_1_**–**C_3_:** Treated cells with 25 µg/mL of PSC-IP; **D_1_**–**D_3_:** Treated cells with 50 µg/mL of PSC-IP.

Morphological examination of cells with collagen solutions could also reveal its biocompatibility and non-toxicity [2,4]. For example, 3T3 F442A cells were treated collagen solutions from jellyfish and there were no observable changes among cells when compared with control group [2]. In the present study, NIH-3T3 cells were treated with PSC-IP (12, 25, and 50 μg/mL) and examined for morphological changes. Treated cells showed no significant change in comparison with the untreated cells, as cells in all groups grew uniformly and presented normal morphologies (Figure 7). The PSC-IP product facilitated the growth of NIH-3T3 cells, which is in agreement with our MTT results. This phenomenon also suggests that collagen from bigeye tuna skin is non-toxic and has the potential to be utilized in biomaterials, for example as a humectant agent and in wound dressing.

### 2.9. In Vitro Scratch Wound Closure

**Figure 8:**
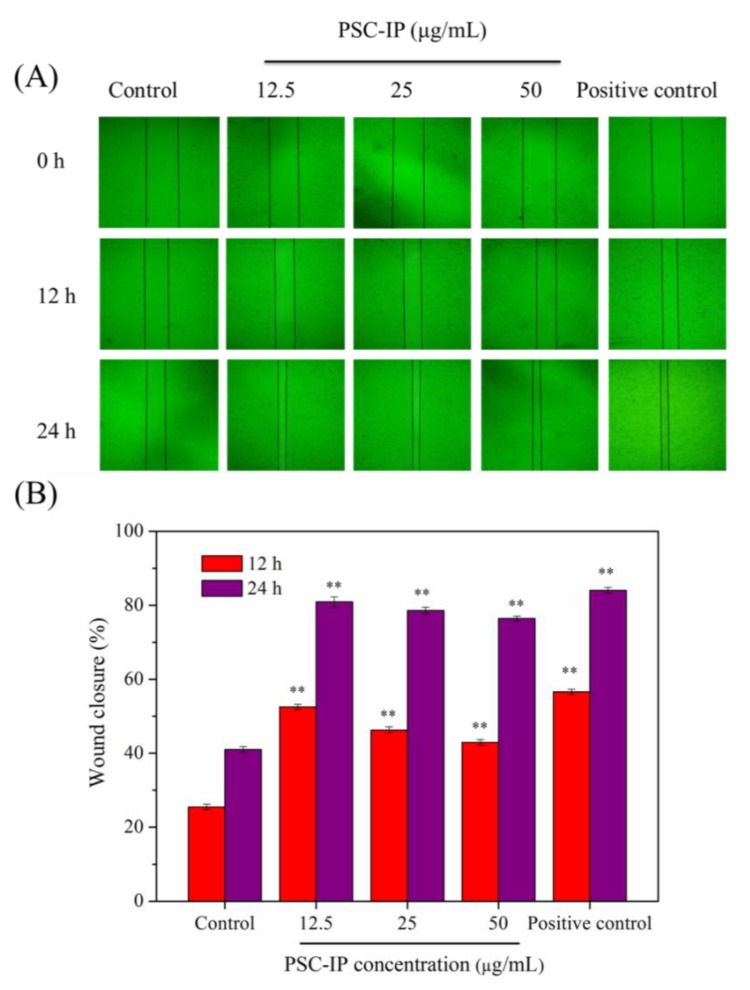
Effects of PSC-IP product from bigeye tuna skin on the scratch closure rate. (**A**) PSC-IP promoted cell migration was evaluated using a scratch wound healing assay. (**B**) Wound closure rate. * *p* < 0.05 and ** *p* < 0.001 vs. control.

PSC extracted from various marine organisms have been shown to be beneficial for wound healing, as they can reinforce the adhesion and proliferation of the fibroblasts, and influence inflammatory cytokines such as IL-1β [35]. Fibroblast migration can accelerate the process of wound re-epithelialization and promote wound closure during wound healing [36]. In the previous studies, in vitro scratch test was often used to simulate wound healing [36,37]. Thus, in our study, the effects of the PSC sample on wound healing were estimated via an in vitro scratch test *(*Figure 8). The scratch closure rate at different time points was recorded and calculated. Figure 8A shows that the wound scratch area decreased remarkably in a dose-dependent manner when treated with the PSC-IP product in comparison with the negative control group area. In addition, the scratch closure rates of the PSC-IP product treated groups were apparently greater than the control group (Figure 8B). It is worth noting, that the scratch nearly closed in the experimental groups, and showed a stronger healing effect similar to bovine collagen after 24 h of treatment. Overall, in vitro wound healing results indicate that collagen from bigeye tuna skin can effectively promote cell migration and has the potential for wound healing. However, the molecular mechanism of collagen promoting cell migration and proliferation is also unclear. In our further study, some biochemical/cellular signaling pathways will be chosen for investigating, such as AKT/mTOR signaling or nuclear factor kappa enhancer binding protein (NF-ĸB) signaling pathway [36]. 

## 3. Materials and Methods

### 3.1. Raw Materials

Bigeye tuna skin was provided by Zhejiang Ocean Family CO., LTD (Zhoushan, China). The NIH-3T3 fibroblasts were stored in the laboratory [4]. Bovine collagen type I (cat. no. C8060) was purchased from Solarbio (Beijing, China). The LDH cytotoxicity assay kit (cat. no. C0016) and the high molecular weight markers (cat. no. P0068) was obtained from Beyotime Biotechnology (Shanghai, China). The MTT cell proliferation and cytotoxicity assay kit (cat. no. AR1156) was obtained from Boster Biological Technology Co., Ltd (Wuhan, China). All other reagents were of analytical grade.

### 3.2. Extraction of Collagen from Bigeye Tuna Using the Salting-Out Method 

The extraction procedure of PSC extraction from bigeye tuna skin was performed at 4 °C according to Tang et al. [4]. Fish skins that had been removed from non-collagenous protein and defatted were cut into small pieces and incubated in 0.5 M acetic acid (1:50, *w/v*) and 1200 U/g pepsin to extract PSC. The extract was then filtered and the supernatants were salted-out using 1.5 M NaCl. After 24 h, the precipitate was harvested by centrifugation (10,000× g, 15 min) and dissolved in acetic acid (0.5 M) subsequently. The PSC was dialyzed with deionized water until the pH was neutral and the silver nitrate method was used to detect the presence of chloride ions. The resultant suspensions (PSC-SO) were lyophilized and stored at −20 °C for further study. 

### 3.3. Extraction of Collagen from Bigeye Tuna by Using Isoelectric Precipitation

The small pieces of fish skins mentioned above were incubated in 0.5 M acetic acid (1:50, *w/v*) and 1200 U/g pepsin to extract PSC. The extract was then filtered and the obtained supernatants were named as collagen stock solution. Then, the pH of collagen stock solution (3 mg/mL) was adjusted to 1.0, 2.0, 3.0, 4.0, 5.0, 6.0, 7.0, 8.0, 9.0, or 10.0 with HCl (6 N) or NaOH (6 N), allowing to stand for approximately 3 h. The supernatant was obtained after centrifugation (10,000 × *g*, 15 min) and used to determine the protein concentration according to the instructions of the BCA protein detection kit. Then, the collagen stock solution was adjusted to the isoelectric point of collagen. The precipitate was harvested by centrifugation (10,000× *g*, 15 min), and dissolved in acetic acid (0.5 M). The samples were dialyzed with deionized water until the pH value was neutral. The resultant suspensions (PSC-IP) were lyophilized and stored at –20 °C for further study.

### 3.4. SDS-PAGE Analysis

The SDS-PAGE method was performed according to Laemmli et al. [38]. Protein (30 μg) obtained from PSC-SO and PSC-IP was loaded in each well of pre-made 8.0% SDS-PAGE gels. The molecular weights of collagen samples were then estimated by inferring from the high molecular weight markers. Bovine collagen type I was used as a positive control. 

### 3.5. Amino Acid Analysis

PSC-SO and PSC-IP products (0.02 g each) were hydrolyzed in 6 N HCl at 110 °C for approximately 24 h. The hydrolysates were diluted and then analyzed using an amino acid analyzer (Hitachi L-8800, Tokyo, Japan). The chloramine T method was used to analyze the content of hydroxyproline [15]. 

### 3.6. FTIR Analysis

FTIR spectra of PSC-IP product were determined using a Bruker Tensor 27 FTIR spectrometer (Bruker, Rheinstetten, Germany) under dry conditions. The infrared spectra were recorded in the 4000–500 cm^–1^ range at 1 cm^–1^ resolution for a single scan.

### 3.7. ICP-MS 

The contents of heavy metals in the PSC-IP product were analyzed using ICP-MS (Agilent, CA, USA). The PSC-IP (0.5 mg/mL) was dissolved in deionized water and cooled to room temperature before detection [14]. 

### 3.8. Cytotoxic and Allergenic Properties of PSC-IP

NIH-3T3 fibroblasts were used to determine the cytotoxic and allergenic properties of PSC-IP product according to the MTT assay kit instructions. Cells were inoculated in 96-well plates (1 × 10^5^ cells/well) and incubated in a 5% CO_2_ incubator for 24 h at 37 °C. Then, cells were dealt with PSC-IP (0, 6.25, 12.5, 25, 50 and 100 μg/mL) and cultured for another 24 h. The cytotoxic possibility of the PSC-IP product was determined by MTT assay and the absorbance values were determined at 490 nm. Cell growth inhibition (%) was obtained according to the MTT assay kit instructions.

The allergenic properties of the PSC-IP product were determined using the LDH release assay. Cells were inoculated in 96-well plates (1 × 10^5^ cells/well) and cultured for 24 h. The cells were dealt with PSC-IP (0, 6.25, 12.5, 25, 50, and 100 μg/mL) and cultured for another 24 h. The LDH release rate (%) was calculated according to the LDH cytotoxicity assay kit instructions.

### 3.9. Morphological Changes

NIH-3T3 fibroblasts (1 × 10^5^ cells/mL) were suspended and cultured in a 6-well flat bottom plate with a cover glass (20 × 24 mm) for 24 h. Cells were dealt with PSC-IP (0, 12.5, 25, and 50 μg/mL). After 24 h incubation, the changes in cell morphology were assessed using an inverted microscope (Olympus, Tokyo, Japan).

### 3.10. In Vitro Scratch Closure Assay 

NIH-3T3 cells were inoculated into 6-well plates (2 × 10^5^ cells/well) and cultured for 24 h at 37 °C in 5% CO_2_ to reach 80–90% cell confluency. A scratch wound was created using a 200 μL pipette tip and the wound debris was washed away using PBS. PSC-IP product (0, 12.5, 25, and 50 µg/mL) was added and cultured for a further 12 or 24 h. The phase-contrast microscope (CKX41-A32PH, Olympus, Tokyo, Japan) was used to observed the scratch closure and the scratch area was obtained using Image J software. 

The scratch closure rate (%) was calculated as follows:

Scratch closure rate (%) = (A_0_ − A_t_)/A_0_ × 100%

where A_0_ represents the scratch area at 0 h and A_t_ represents the scratch area at the designated time point.

### 3.11. Statistical Analysis

All tests were expressed as mean ± standard deviation (SD, *n* = 3). Data were analyzed by analysis of variance (ANOVA) using IBM SPSS 19.0 software (Ehningen, Germany). A difference was considered statistically significant when * *p* < 0.05 and ** *p* < 0.001. 

## 4. Conclusions

In the present study, PSC was extracted from bigeye tuna skin using salt outing (PSC-SO) and isoelectric precipitation (PSC-IP) methods. Considering the high yield and short time for extracting PSC, the isoelectric precipitation method was chosen for obtaining PSC-IP product from bigeye tuna skin. SDS-PAGE analysis indicated that PSC-IP product from bigeye tuna is a collagen type I. Furthermore, ICP-MS analysis showed that the PSC-IP product was free of heavy metals. The effects of the PSC-IP on proliferation and migration PSC-IP indicates that collagen from bigeye tuna skin has good potential for use in cosmetic and biomedical applications. 

## Figures and Tables

**Table 1:** Elemental analysis of PSC-IP product from bigeye tuna skin by ICP-MS.

Collagen	Element	Content (mg/kg)	National Standard of Edible Gelatin (GB 6783-2013 in China) (mg/kg)
PSC-IP	As	0.51 ± 0.04	≤1.0
	Pb	0.17 ± 0.02	≤1.5
	Hg	1.18 ± 0.07	
